# Antagonism between Gdf6a and retinoic acid pathways controls timing of retinal neurogenesis and growth of the eye in zebrafish

**DOI:** 10.1242/dev.130922

**Published:** 2016-04-01

**Authors:** Leonardo E. Valdivia, Dayna B. Lamb, Wilson Horner, Claudia Wierzbicki, Amanuel Tafessu, Audrey M. Williams, Gaia Gestri, Anna M. Krasnow, Terra S. Vleeshouwer-Neumann, McKenzie Givens, Rodrigo M. Young, Lisa M. Lawrence, Heather L. Stickney, Thomas A. Hawkins, Quenten P. Schwarz, Florencia Cavodeassi, Stephen W. Wilson, Kara L. Cerveny

**Affiliations:** 1 Department of Cell and Developmental Biology, UCL, Gower Street, London WC1E 6BT, UK; 2 Department of Biology, Reed College, 3203 SE Woodstock Boulevard, Portland, OR 97202, USA

**Keywords:** Retinoic acid, BMP, Gdf6a, Ciliary marginal zone, Neurogenesis, Retinal stem cells, Zebrafish

## Abstract

Maintaining neurogenesis in growing tissues requires a tight balance between progenitor cell proliferation and differentiation. In the zebrafish retina, neuronal differentiation proceeds in two stages with embryonic retinal progenitor cells (RPCs) of the central retina accounting for the first rounds of differentiation, and stem cells from the ciliary marginal zone (CMZ) being responsible for late neurogenesis and growth of the eye. In this study, we analyse two mutants with small eyes that display defects during both early and late phases of retinal neurogenesis. These mutants carry lesions in *gdf6a*, a gene encoding a BMP family member previously implicated in dorsoventral patterning of the eye. We show that *gdf6a* mutant eyes exhibit expanded retinoic acid (RA) signalling and demonstrate that exogenous activation of this pathway in wild-type eyes inhibits retinal growth, generating small eyes with a reduced CMZ and fewer proliferating progenitors, similar to *gdf6a* mutants. We provide evidence that RA regulates the timing of RPC differentiation by promoting cell cycle exit. Furthermore, reducing RA signalling in *gdf6a* mutants re-establishes appropriate timing of embryonic retinal neurogenesis and restores putative stem and progenitor cell populations in the CMZ. Together, our results support a model in which dorsally expressed *gdf6a* limits RA pathway activity to control the transition from proliferation to differentiation in the growing eye.

## Abstract (precis)

**Summary:** In the vertebrate eye, dorsally expressed Gdf6a limits RA pathway activity to control the transition from proliferation to differentiation, thereby regulating eye size.

## INTRODUCTION

The balance between cell proliferation and differentiation is spatially and temporally regulated during development, ensuring the generation of tissues with the correct proportion of differentiated cells (Schmidt et al., 2013; Urbán and Guillemot, 2014). In the vertebrate retina, this process begins when retinal progenitor cells (RPCs) successively exit the cell cycle to generate retinal ganglion cells (RGCs), cone photoreceptors, interneurons, rod photoreceptors and Müller glia (Cepko, 2014). This progression of fate specification and the timing of neurogenic decisions require cells to integrate intrinsic information and extrinsic signals (Cepko, 2014; Cerveny et al., 2012). Cell cycle regulators and a variety of transcription factors are implicated in the cell-autonomous progression from RPC to post-mitotic neuron, and secreted signals can influence the transition from proliferation to differentiation (Agathocleous and Harris, 2009; Bassett and Wallace, 2012).

In vertebrate eyes, neurogenesis proceeds in waves. In zebrafish, neuronal differentiation spreads across the developing retina from a ventronasal patch of RPCs cells adjacent to the optic stalk (Rappaport, 2006). This spread of neurogenesis is preceded by comparable waves of expression of neurogenesis-related genes including the proneural gene *atoh7* and the paired-class cone-rod homeobox gene *crx* (Masai et al., 2000; Shen and Raymond, 2004). Differentiation of distinct neuronal types continues to propagate circumferentially in successive waves, ultimately filling the central retina by 48 hours post fertilisation (hpf). After this point, neurogenesis occurs radially, with new neurons added to the eye in successive rings from a peripheral area of the retina termed the ciliary marginal zone (CMZ). The CMZ contains a stem cell niche that allows continued growth and neurogenesis in the eye (Centanin et al., 2011; Stenkamp, 2007).

Retinal neurogenesis occurs in a context where extrinsic signals impart nasotemporal and dorsoventral (DV) positional identity to newly generated neurons. In fish, the opposing actions of Fgf and Shh initiate nasotemporal patterning (Hernandez-Bejarano et al., 2015) whereas opposing Bmp and Hedgehog (Hh) signals (Yang, 2004) establish DV identities. For instance, Gdf6a (BMP13 homologue) and other BMPs are expressed in dorsal domains and influence dorsal identity within the retina (French et al., 2009; Gosse and Baier, 2009; Kruse-Bend et al., 2012; Williams et al., 2008). Mutations in *gdf6* genes have also been linked to other ocular anomalies, including microphthalmia and coloboma (Asai-Coakwell et al., 2013; den Hollander et al., 2010; French et al., 2009). It is likely that the connection between axial patterning signals and positional identity is maintained within the CMZ as newly generated RGCs must integrate into existing circuitry and project to topographically appropriate target regions within the brain.

The RA signalling pathway also influences retinal development. RA-synthesising enzymes are expressed predominantly in the ventral retina and RA signalling is required for morphogenesis of the ventral eye, including choroid fissure closure (Lupo et al., 2011; Marsh-Armstrong et al., 1994). Expression of the RA synthesis enzyme *aldh1a3* is expanded dorsally in eyes of *gdf6a* morphant embryos (French et al., 2009) raising the possibility that interactions between RA and Gdf6 signals occur during retinal development.

In this study, we sought to identify signals that regulate eye growth and found that, in addition to their roles in DV patterning, opposing Gdf6a and RA signals influence proliferation and differentiation of RPCs. Through a forward genetic screen in zebrafish, we identified two point mutations in *gdf6a* that lead to reduction in size of the CMZ and subsequent small eye phenotypes. These phenotypes are accompanied by an expansion of RA pathway activity within the CMZ and precocious differentiation of RPCs. We find that activating the RA pathway in wild-type eyes phenocopies *gdf6a* mutants and that abrogation of RA signalling ameliorates the *gdf6a* mutant phenotype. Together, our results show that signals that impart DV identity in the eye also regulate production of neurons and retinal growth, revealing an unappreciated link between the pathways that regulate patterning, proliferation, and stem/progenitor cell maintenance in the developing eye.

## RESULTS

### *gdf6a* mutants exhibit small eyes independent of apoptosis

**Fig. 1.**
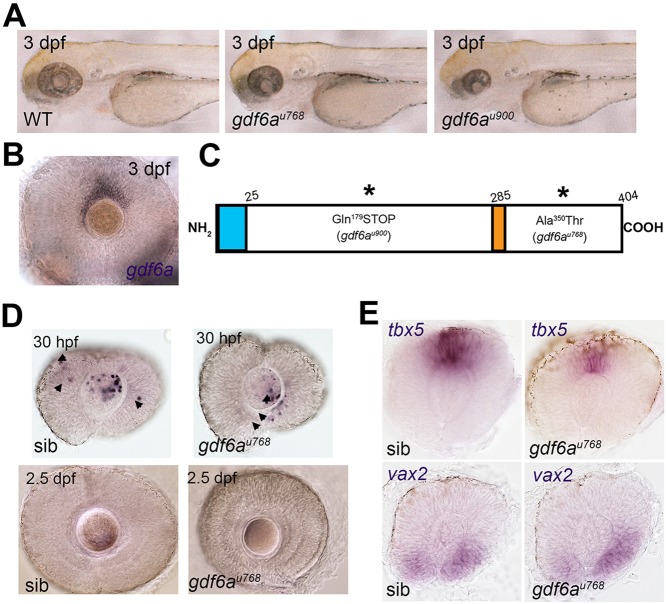
**Small-eye phenotype in *gdf6a* mutants is independent of programmed cell death in developing retina.** (A) Lateral views of wild-type and *gdf6a* mutants showing small eyes. (B) Lateral view of dorsal *gdf6a* expression (purple) in wild-type retina. (C) Domain structure of Gdf6a protein indicating the N-terminal signal peptide (amino acids 1-25, light blue), the furin protease recognition site (amino acids 280-285, orange), and the premature stop codon of *gdf6a^u900^* and missense mutation (Ala350Thr) in *gdf6a^u768^* (asterisks). (D) Lateral views of eyes of siblings (sib) and *gdf6a* mutants. Similar levels of TUNEL^+^ apoptotic cells (purple) are evident in the *gdf6a^u768^* and sibling eyes at 30 hpf; most apoptotic cells are in the lenses (arrows) and only few in the retinae (arrowheads). TUNEL^+^ cells are undetectable in eyes of either genotype by 2.5 dpf (lower panels). (E) Lateral views of whole-mount eyes of 24 hpf *gdf6a^u768^* and sibling embryos showing expression (purple) of markers for dorsal (*tbx5a*) and ventral (*vax2*) retinal character.

Clutches of 72 hpf embryos from families of zebrafish carrying N-ethyl-N-nitrosurea (ENU)-induced mutations (Valdivia et al., 2011) were examined for ocular abnormalities, including microphthalmia. We recovered two non-complementing, recessive mutations, u768 and u900, that when homozygous led to small eyes with ventrally displaced lenses and, in some cases, coloboma (Fig. 1A). Although both homozygous mutant phenotypes are fully penetrant, u900 mutants consistently display smaller eyes than u768 mutants (Fig. S1D).

Both mutants carry lesions in *gdf6a*, a gene that is expressed dorsally both in the developing retina (French et al., 2009; Gosse and Baier, 2009) and the CMZ (Fig. 1B). Using bulk segregant analysis, complementation testing, and sequencing, we identified u768 as a missense mutation that results in the substitution of a threonine for an absolutely conserved alanine at position 350 in the Gdf6a protein (Fig. 1C). It is likely that this mutation generates a hypomorphic Gdf6a protein. Sequencing identified u900 as a nonsense mutation that converts the codon for Gln179 to a Stop (*gdf6a^u900^*; Fig. 1C). This mutation is predicted to generate a truncated protein lacking the conserved carboxy-terminal signalling domain.

Previous studies have shown that loss of Gdf6a function is associated with a transient wave of retinal apoptosis, suggesting that Gdf6a is required for RPC survival (den Hollander et al., 2010; Gosse and Baier, 2009). However, in *gdf6a^u768^* mutants, the number of apoptotic retinal cells between 24 and 36 hpf was similar to wild type [*gdf6a^u768^*=5.25±2.75 (mean±s.d.); wild-type siblings=3.4±1.76; *P*=0.12; *n*=13 embryos of each genotype; Fig. 1D; Fig. S1A], and retinal neurons appeared healthy, suggesting that cell death is not the main driver of the small eye phenotype in this mutant. Although *gdf6a^u900^* mutants displayed increased levels of retinal apoptosis at around 30 hpf (Fig. S1B), blocking cell death with the pan-caspase inhibitor Z-VAD-FMK reduced apoptotic levels but did not rescue the subsequent small eye size, corroborating previously published data (French et al., 2013) (Fig. S1C).

Functional studies have also implicated Gdf6a in patterning the DV axis of the retina (French et al., 2009; Gosse and Baier, 2009; Rissi et al., 1995). However, the u768 allele exhibits overtly normal expression of DV markers at 24 hpf (Fig. 1E) suggesting that the small eye phenotype arises in this mutant allele in the absence of major defects in the establishment of axial patterning of the retina. Together, these data suggest that a mechanism other than programmed cell death and/or abnormal retinal patterning is responsible for the small eye phenotype in *gdf6a* mutants.

### The CMZ is reduced in *gdf6a* mutants

**Fig. 2.**
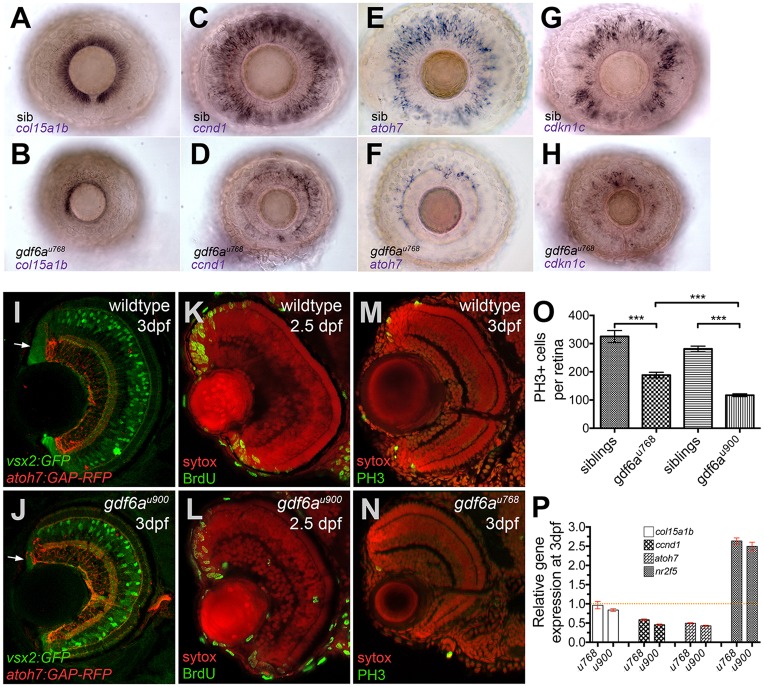
***gdf6a* mutants exhibit decreased expression of stem, progenitor and committed cell genes and reduced proliferation within the CMZ.** (A-H) Lateral views of whole-mount eyes from 3 dpf embryos (genotype bottom left) showing expression (purple) of various genes (indicated bottom left) in the CMZ. (A,B) *col15a1b*, a marker of the peripheral putative stem cell compartment, is expressed in the sibling CMZ (A), but reduced in the dorsal and nearly absent in the ventral CMZ of the *gdf6a^u768^* eye (B). (C,D) *ccnd1* is highly expressed in proliferating progenitors of wild-type CMZs (C), but reduced in *gdf6a^u768^* mutant eyes (D). (E-H) *atoh7* and *cdkn1c* are expressed in committed precursors in wild-type CMZs (E,G) but in the *gdf6a^u768^* mutant eyes they are strongly downregulated (F,H). (I,J) Transverse sections of 3 dpf sibling and *gdf6a^u900^* eyes carrying the *vsx2:GFP* (green) and *atoh7:GAP-RFP* (red) transgenes highlighting a reduced CMZ in the *gdf6a^u900^* mutant (white arrows). (K-N) Coronal sections immunostained for markers of proliferation (green; BrdU incorporation in K,L or PH3 in M,N) and then counterstained with SYTOX Orange (red nuclei). (O) Graph showing numbers of PH3^+^ mitotic retinal cells in eyes of *gdf6a* mutant and sibling embryos. All PH3^+^ cells in ten whole-mount 60 hpf eyes were counted and graphed with standard error bars (95% confidence limits; Student's *t*-test, ***P≤0.0004). (P) Graph showing real-time PCR quantification of gene expression changes of *col15a1b*, *ccnd1*, *atoh7* and *nr2f5* in 3 dpf dissected mutant and wild-type retinas normalised to *β-actin*. Wild-type values for each gene were set to 1 and mutant fold changes were plotted relative to this value (±s.e.).

As the CMZ is the primary source of new neurons and retinal growth in fish larvae (Centanin et al., 2011; Raymond et al., 2006), we examined expression of markers for subdomains of the CMZ in *gdf6a* mutants and found striking differences compared with wild-type eyes as early as 2.5 days post fertilisation (dpf). The most peripheral *col15a1b*-expressing region, which contains presumptive stem cells (Cerveny et al., 2010; Gonzalez-Nunez et al., 2010), is reduced in *gdf6a^u768^* mutants with fewer *col15a1b*-positive cells in the dorsal CMZ and virtually no *col15a1b* expression in the ventral CMZ (Fig. 2A,B). Similarly, all other subdomains of this germinal zone – proliferating progenitors (expressing *ccnd1*), specified precursors (expressing the transcription factor *atoh7*), and differentiating neurons (expressing the cyclin-dependent kinase inhibitor *cdkn1c*) (Cerveny et al., 2010) – are reduced (Fig. 2C-H). Similarly, altered patterns of gene expression were observed in the CMZ of *gdf6a^u900^* eyes (Fig. S2).

To characterise the mutant CMZs further, we examined expression of *Tg[vsx2:GFP]*. *vsx2* encodes a homeodomain transcription factor expressed throughout the CMZ, in Müller glia and in a subset of bipolar cells (Vitorino et al., 2009). Consistent with fewer cells within the CMZ, we observed a smaller *vsx2*:GFP-expressing region with only a few cells separating the retinal margin and *atoh7:GAP-RFP*-expressing RGCs in 60 hpf *gdf6a^u900^* mutant retinae (Fig. 2I,J).

Consistent with decreased growth from a smaller CMZ, *gdf6a* mutant eyes contained fewer proliferative cells in both S phase [marked by bromodeoxyuridine (BrdU) staining] and M phase [marked by phospho-histone H3 (PH3) staining] of the cell cycle (Fig. 2K-N) by 2.5 and 3 dpf (Fig. 2O; *n*=10 embryos for each genotype). These findings indicate that reduced Gdf6a function is correlated with decreased cell proliferation in the CMZ.

To gauge expression levels of CMZ markers quantitatively, we used real-time PCR (qPCR) in dissected mutant and wild-type sibling larval retinae. Both mutant alleles show nearly identical reduction in transcript levels of genes expressed in proliferating progenitors (*ccnd1*) and committed precursors (e.g. *atoh7*) (Fig. 2P). Although *in situ* hybridisation clearly shows fewer *col15a1b*-positive cells, qPCR detected only a modest reduction in transcript levels. Not all genes analysed, however, showed decreased expression. A control gene, *nr2f5*, encoding a transcriptional target of RA signalling (Love and Prince, 2012) was elevated nearly 2.5-fold in *gdf6a* mutant eyes (Fig. 2P), suggesting increased RA pathway activity.

### *gdf6a* mutants display RA pathway activity throughout the peripheral CMZ

**Fig. 3.**
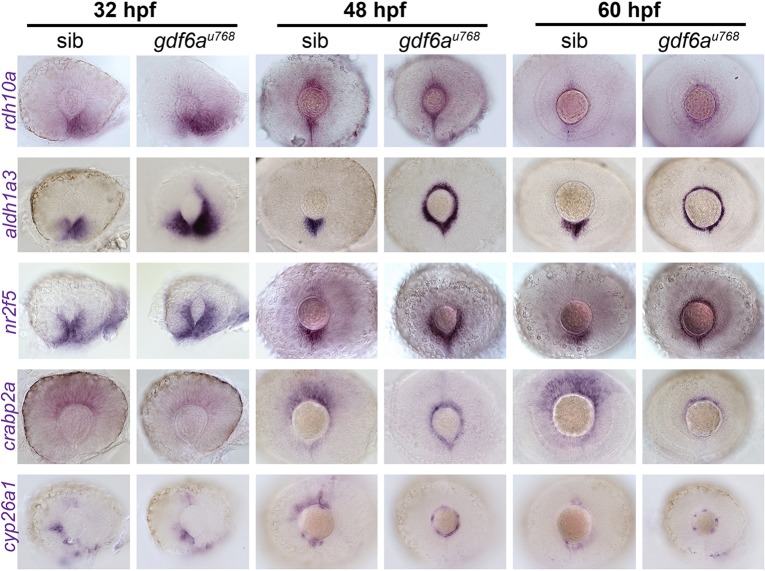
**Expression of genes encoding RA pathway components is expanded dorsally in the CMZ of *gdf6a* mutants.** Lateral views of whole-mount eyes from 32, 48 and 60 hpf embryos showing expression (purple) of the genes indicated to the left of the rows. Genes shown encode enzymes required for RA synthesis (*rdh10a* and *aldh1a*3), a direct transcriptional target of the RA pathway (*nr2f5*), a cytosolic RA-buffering protein (*crabp2a*) and an enzyme responsible for RA catabolism (*cyp26a1*).

RA synthesis and target genes are ectopically activated throughout the circumference of the peripheral-most compartment of the CMZ of *gdf6a* mutants. Expression of two genes involved in RA synthesis, retinol dehydrogenase (*rdh10a*) and a retinaldehyde dehydrogenase (*aldh1a3*), is increased and expanded dorsally in the *gdf6a^u768^* mutants (Fig. 3, top two rows). Consequently, and as expected from qPCR experiments, *nr2f5* is transcribed in more cells of the presumptive CMZ (Fig. 3, middle row). Consistently, *gdf6a^u768^* mutants carrying an RA-responsive YFP transgene (Perz-Edwards et al., 2001) exhibit expanded YFP expression in the peripheral compartment of the CMZ (Fig. S3). Genes likely to limit RA activity, such as the cytosolic RA binding protein *crabp2a* (Cai et al., 2012) and the RA-degrading enzyme *cyp26a1* (Isken et al., 2008), are expressed in fewer cells in *gdf6a* mutant eyes (Fig. 3, bottom two rows). Together, these data indicate that RA signalling is enhanced in the CMZ of *gdf6a* mutants.

### Enhanced RA pathway activity inhibits eye growth

**Fig. 4.**
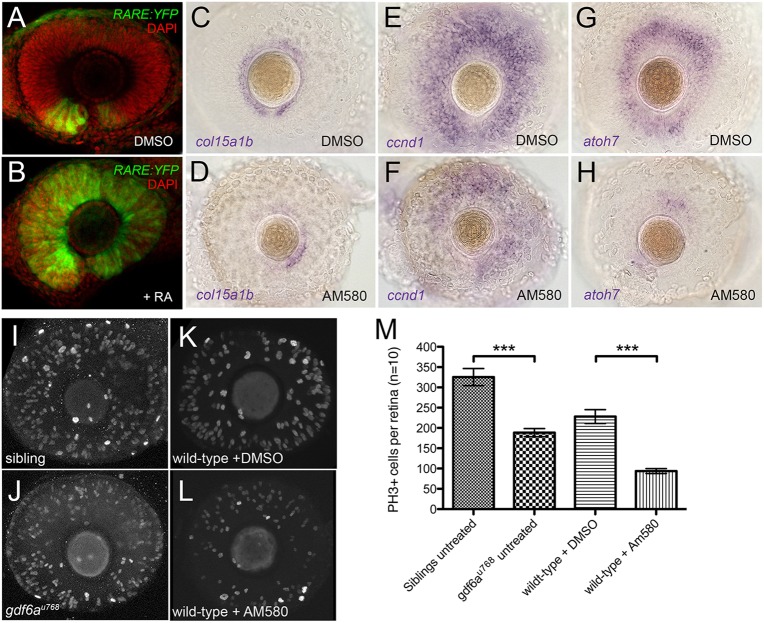
**Pharmacological activation of the RA pathway in wild-type embryos mimics the *gdf6a* mutant eye phenotype.** (A,B). Representative lateral views of maximum intensity projections of eyes from *Tg[RARE:YFP]* embryos immunostained for GFP (*RARE:YFP*, green) and counterstained with DAPI (red). Eyes are from embryos that were incubated in vehicle control (DMSO; A) or in 13-cis RA (B), exposed to a pulse of UV light, and then fixed and immunostained at 33 hpf. The majority of RPCs in B have responded to RA by activating the *RARE:YFP* transgene. (C-H) Lateral views of eyes from wild-type embryos treated with vehicle (DMSO; C,E,G) or the RAR agonist AM580 (25 nM; D,F,H) from 24 to 36 hpf and analysed at 60 hpf for expression of CMZ genes *col15a1b* (C,D), *ccnd1* (E,F) and *atoh7* (G,H). (I-L) Representative lateral views of maximum intensity projections of 60 hpf embryonic eyes immunostained for PH3. Eyes from an untreated sibling and untreated *gdf6a^u768^* mutant (I and J, respectively) and from a DMSO-treated wild type and an AM580-treated wild type (K and L, respectively) are shown. (M) Graph quantifying PH3^+^ cells in I-L. The average number of PH3^+^ cells per retina (*n*=10 embryos) was calculated and then plotted with standard error bars (95% confidence limits; pair-wise comparisons using Student's *t*-test, ***P≤0.0001).

We next asked whether excessive RA affects eye size. We first tested whether all wild-type retinal progenitors respond to RA. Embryos carrying the *Tg[RARE:YFP]* RA reporter transgene (Perz-Edwards et al., 2001) were incubated with 13-cis RA, which can be locally converted to all-trans RA by UV-driven photo-isomerisation to activate the RA pathway (Xu et al., 2012). In 24-36 hpf wild-type embryos, only ventral retinal cells activate the RA pathway reporter transgene in the absence of exogenous all-trans RA (Fig. 4A). UV-mediated activation of RA signalling, however, demonstrated that most, if not all, RPCs are capable of responding to RA (Fig. 4B). These data suggest that endogenous RA signalling is spatially limited in the eye by RA availability, not cell competence to respond to RA.

We next investigated whether pharmacological activation of the RA pathway using AM580, a retinoic acid receptor alpha (RARα) agonist (Gianni et al., 1996), affects eye development similarly to *gdf6a* mutants. Embryos incubated in AM580 consistently displayed smaller eyes with fewer PH3^+^ cells (Fig. 4I-M). Pairwise *t*-test comparisons indicate that both *gdf6a* mutant and AM580-treated embryos contain significantly fewer PH3^+^ cells than sibling or DMSO-treated wild-type embryos (Fig. 4M). Permutation testing of relative means (untreated *gdf6a^u768^* mutants/untreated siblings and AM580-treated/DMSO-treated wild types) revealed that the proportion of PH3^+^ cells in *gdf6a^u768^* mutants relative to the number of PH3^+^ cells in sibling eyes is not statistically different from the proportion of PH3^+^ cells in AM580-treated eyes relative to wild type (*P*=0.56; Fig. S4). In addition, RA pathway upregulation is correlated with reduced expression of markers for putative stem and progenitor cells within the CMZ (Fig. 4C-H).

### Inhibition of the RA pathway restores the CMZ in *gdf6a* mutants

**Fig. 5.**
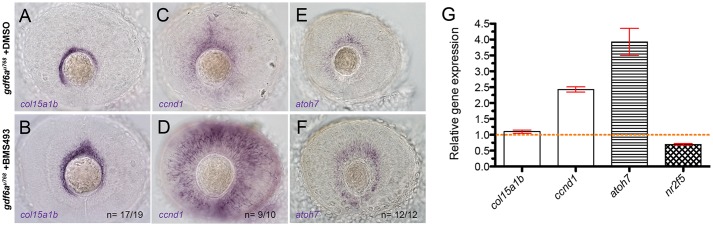
**Pharmacological inhibition of the RA pathway partially rescues the *gdf6a* mutant CMZ phenotype.** (A-F) Lateral views of eyes from 60 hpf *gdf6a^u768^* mutants treated with vehicle (DMSO;A,C,E) or the RAR inverse agonist BMS493 (15 µM; B,D,F) showing expression of *col15a1b* (A,B), *ccnd1* (C,D) and *atoh7* (E,F). The number of embryos with staining pattern similar to that shown is indicated as a fraction in lower right corner of images. (G) qPCR quantification of relative gene expression levels for *col15a1b*, *ccnd1*, *atoh7* and *nr2f5* in 60 hpf mutant eyes and eyes from mutants treated with BMS493, normalised to *β-actin*. Values for each gene from DMSO-treated mutant eyes were set to 1 and fold changes in BMS493-treated eyes were plotted relative to this value (±s.e.).

To evaluate the extent to which misregulation of RA pathway activity contributes to the imbalance in proliferation and differentiation, and subsequent growth of *gdf6a* mutant eyes, we asked whether reducing RA pathway activity could restore the CMZ in *gdf6a* mutant retinae. When clutches of embryos from a *gdf6a^u768^* carrier in-cross were incubated with the panRAR inverse agonist BMS493 (Germain et al., 2009), mutant eyes contained more cells expressing *col15a1b*, *ccnd1* and *atoh7* in the peripheral retina (Fig. 5A-F). We also quantified transcript levels for CMZ markers in BMS493-treated eyes relative to DMSO-treated eyes and confirmed *in situ* hybridisation data that *ccnd1* and *atoh7* levels were increased (Fig. 5G). Because changes in *col15a1b* detected by qPCR were modest, we measured the percentage area of *col15a1b*-positive cells in control and BMS-treated eyes and found that treated eyes contained a nearly 40% larger *col15a1b*-positive area than did controls (Fig. S5C). Sibling eyes with the same treatment exhibited a noticeable expansion of *col15a1b* in the ventral retina (Fig. S5B). Together, these data suggest that RA limits expression of CMZ markers in *gdf6a* mutants and contributes to CMZ maintenance during normal development.

### Retinal precursors precociously generate neurons in *gdf6a* mutants

**Fig. 6.**
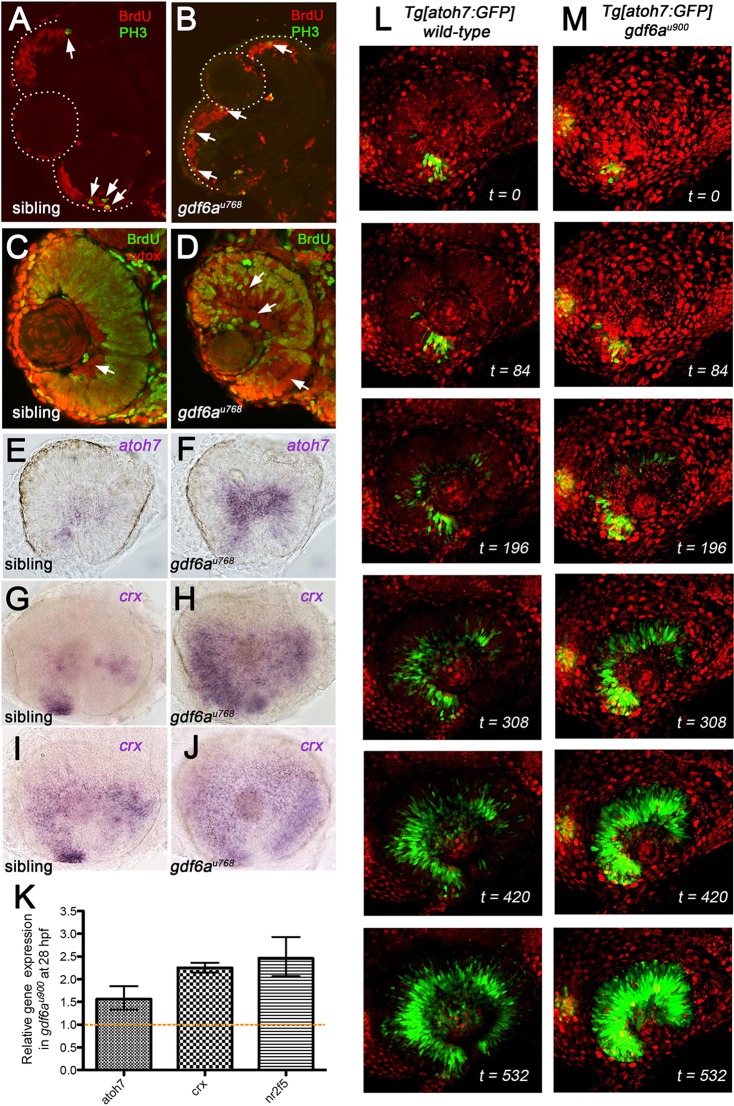
***gdf6a* mutant retinae exhibit accelerated differentiation of neurons.** (A,B) Transverse cryosections of retinae from sibling (A) and *gdf6a^u768^* (B) embryos injected with BrdU twice at 56 hpf, again at 58 hpf, and then fixed at 60 hpf and immunostained for markers of DNA replication (BrdU, red) and G2/M phase (PH3, green). Dashed lines demarcate the lens and retinal boundaries. White arrows point to double-labelled cells. (C,D) Transverse cryosections of retinae from sibling (C) and *gdf6a^u768^* (D) embryos injected with BrdU at 50 hpf, fixed at 60 hpf, and then immunostained for BrdU (green) and counterstained with SYTOX Orange (red). White arrows point to regions where neurons were postmitotic at the time of BrdU incubation; in the wild-type eye, these are restricted mostly to the RGC layer but are more widely present in the mutant eye. (E-J) Lateral view of whole-mount eyes from sibling and mutant embryos showing expression (purple) of *atoh7* at 28 hpf (E,F), and *crx* at 36 hpf (G,H) and 48 hpf (I,J). (K) Real-time PCR quantification of *atoh7*, *crx* and *nr2f5* expression from 28 hpf dissected mutant and wild-type retinas normalized to *β-actin*. Wild-type values for each gene were set to 1 and mutant fold changes were plotted relative to this value (±s.e.). (L,M) Maximum intensity projections at single time points from time-lapse analyses of the generation of *Tg[atoh7:GFP]*-positive RGCs in wild-type (L) and *gdf6a* mutant (M) eyes. Stacks (80 µm) of 2-µm optical sections were captured every 18 min for 9 h from 28.5 hpf (t=0). Nuclei are labelled with histone H2B-mcherry (red). See also Movies 1 and 2. Time (t) is in minutes.

The reduced CMZs in *gdf6a* mutants could result from changes in cell cycle kinetics within the CMZ and/or altered timing of cell cycle exit and transition of post-mitotic cells into the mature retina. To distinguish between these possibilities, all S-phase cells were first labelled with BrdU for 4 h prior to fixation. Next, BrdU incorporation in cells that had progressed to M phase (PH3^+^) was evaluated. Wild-type progenitors can progress from S-phase to M-phase in as little as 2.5 h (Cerveny et al., 2010), and so this labelling regime results in 100% of PH3^+^ cells being labelled by BrdU. In both wild-type sibling and *gdf6a^u768^* mutant eyes, we found that all PH3^+^ cells were also BrdU^+^ (Fig. 6A,B; *n*=16 eyes), suggesting that cell cycle kinetics were not dramatically changed in mutant CMZ.

RPCs exit the cell cycle and differentiate in a stereotypical pattern (Cepko et al., 1996; Harris, 1997), with proliferative RPCs ultimately restricted to the CMZ by ∼2.5 dpf (Stenkamp, 2007). To gauge the balance between proliferation and differentiation after the onset of retinal neurogenesis in *gdf6a* mutants, we labelled all S-phase cells with BrdU for 10 h beginning at ∼50 hpf. With this labelling regime, only a small proportion of cells in wild-type eyes (primarily RGCs) are BrdU negative and are likely to have exited the cell cycle prior to BrdU treatment (Fig. 6C, red cells; Ohnuma et al., 1999). By contrast, all layers of *gdf6a* mutant retinae contained a much larger proportion of BrdU-negative cells (Fig. 6D, white arrows). We observed this striking pattern of precocious cell cycle exit and differentiation in all embryos (*n*=60 total genotyped embryos; 14 *gdf6a^u768^* mutants). These data indicate that the pace of neurogenesis is accelerated in *gdf6a* mutant retinae.

To characterise the precocious production of neurons in *gdf6a* mutants, we analysed the onset and progression of expression of *atoh7* and *crx*, which both exhibit dynamic, fan-shaped ventral-nasal to dorsal-temporal patterns of expression (Masai et al., 2000; Neumann and Nuesslein-Volhard, 2000; Shen and Raymond, 2004). By ∼28 hpf, *atoh7* is expressed in a small patch of ventral RPCs in wild-type eyes (Fig. 6E) whereas a larger proportion of ventral and central RPCs express *atoh7* in *gdf6a* mutant eyes (Fig. 6F). Likewise, expression of *crx*, which is initially transcribed in the ventral-nasal and ventral-central retina at ∼36 hpf in wild-type eyes (Fig. 6G), is more broadly expressed in *gdf6a* mutant eyes (Fig. 6H), similar to wild-type *crx* expression at 48 hpf (compare Fig. 6H and 6I). Quantitative PCR in *gdf6a^u900^* mutant retinas corroborated the upregulation of *atoh7* and *crx* (1.5- and 2-fold greater, respectively), as well as of the RA-target gene *nr2f5* (2.5-fold greater) (Fig. 6K).

To explore the dynamic production of *atoh7*-positive neurons in *gdf6a* mutants, we imaged living *Tg[atoh7:GFP]* embryos. Initiation of GFP fluorescence occurred at the same time in *gdf6a^u900^* mutants and siblings (Fig. 6L,M, top panels), but the number of GFP-expressing cells increased more rapidly and RGC axons extended earlier in mutant retinae (Fig. 6L,M, lower panels; Movies 1, 2), supporting the idea of expedited differentiation in *gdf6a* mutants.

### Precocious RA-mediated differentiation depletes retinal progenitors

**Fig. 7.**
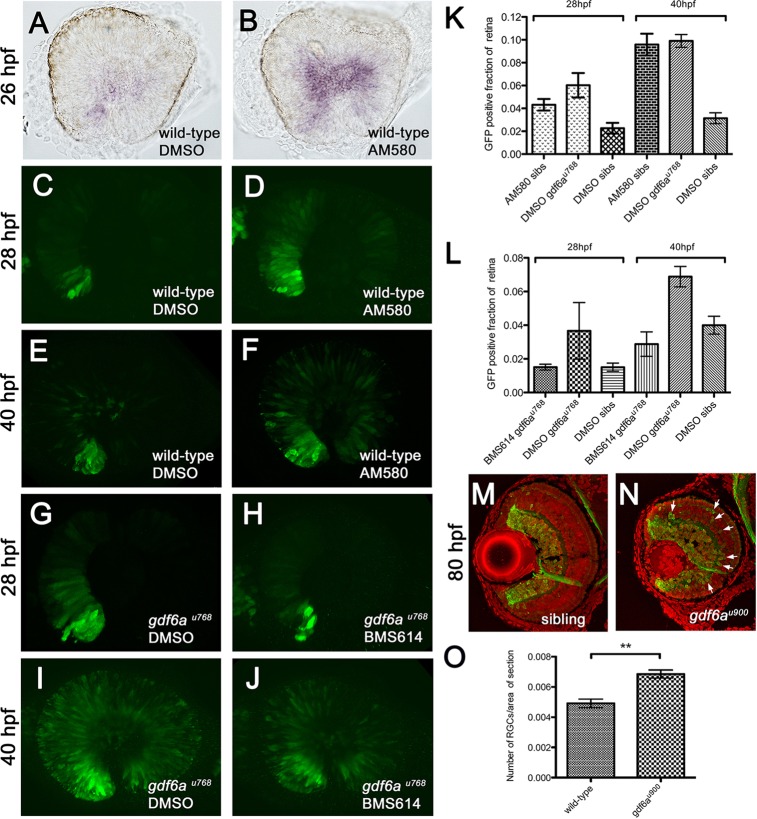
**RA pathway activation accelerates retinal neurogenesis.** (A-F) Lateral views of eyes of wild-type (A,B) or *Tg[atoh7:GFP]* (C-F) embryos treated with AM580 (B,D,F) or vehicle control (DMSO; A,C,E) fixed at the times indicated to the left of the rows, and showing expression of either *atoh7* (purple; A,B) or immunostained for GFP (green; C-F). (G-J) Lateral views of eyes of *gdf6a^u768^* mutants expressing the *atoh7:GFP* transgene treated with BMS614 (H,J) or vehicle control (DMSO; G,I), fixed at the times indicated to the left of the rows, and immunostained for GFP (green). Note that GFP expression does not extend beyond its ventronasal initiation site in the BMS614-treated eye at 28 hpf. (K,L) Graphs showing average proportional volume of retina containing *atoh7:GFP*-positive cells at 28 and 40 hpf in the conditions indicated along the *x*-axes for activation (K) and suppression (L) of RA pathway, plotted with standard error bars (95% confidence limits; *n*=5 embryos for each condition). (M,N) Transverse cryosections of wild-type sibling (M) and *gdf6a^u900^* (N) eyes at 80 hpf showing expression of the *atoh7:GFP* transgene (green) and counterstained nuclei with DAPI (red). White arrows show ectopic GFP-positive cells in the inner nuclear layer. (O) Graph of the average number of *atoh7:GFP*-positive cells per area of retina in wild-type and *gdf6a^u900^* mutant eyes (*n*=6; standard error bars, 95% confidence limits; Student's *t*-test **P=0.0014).

Because RA pathway activity is enhanced in *gdf6a* mutant retinae, we examined whether RA could modulate cell cycle exit and neurogenesis of RPCs by pharmacologically activating or inhibiting RA signalling and then examining *atoh7* and *Tg[atoh7:GFP]* expression. Similar to *gdf6a* mutant retinae, wild-type embryos incubated in AM580 exhibited significantly more *atoh7*^+^ cells (Fig. 7A-F). At 28 hpf, ∼2% of the wild-type retinal volume was GFP^+^, whereas it was more than doubled in both *gdf6a* mutant and AM580-treated wild-type retinae (Fig. 7C,D,K). By 40 hpf, 3% of wild-type retinal volume was GFP^+^, but this percentage was nearly tripled in mutant or AM580-treated wild-type eyes (Fig. 7E,F,K). Pair-wise *t*-tests (*n*=5 for each condition and genotype) indicate that AM580-treated wild-type and *gdf6a^u768^* DMSO-treated eyes are not statistically different from one another at 28 hpf (*P*=0.188) or at 40 hpf (*P*=0.77) and that both are different from DMSO-treated wild-type sibling eyes at both developmental stages (28 hpf: *P*=0.017 for DMSO-treated *u768* mutants versus DMSO-treated wild type, *P*=0.012 for AM580-treated wild type versus DMSO-treated wild type; 40 hpf: *P*=0.000003 for DMSO-treated *u768* mutants versus DMSO-treated wild type, *P*=0.0001 for AM580-treated wild type versus DMSO-treated wild type).

Reducing RA signalling in *gdf6a* mutants with the RAR antagonist BMS614 decreased the number of GFP^+^ cells (Fig. 7G-J) such that DMSO-treated siblings and BMS614-treated *gdf6a^u768^* embryos contained nearly equivalent GFP^+^ retinal volumes (Fig. 7G-J,L). Pair-wise *t*-tests (*n*≥7 for each condition and genotype) indicate that BMS614-treated *gdf6a^u768^* and DMSO-treated sibling eyes contain GFP^+^ retinal volumes that are not statistically different from each other at 28 hpf (*P*=0.566) and 40 hpf (*P*=0.229). At 28 hpf, ∼1.5% of the *gdf6a^u768^* retinal volume was GFP^+^, similar to DMSO-treated siblings and less than that of *gdf6a^u768^* untreated eyes (Fig. 7G-H,L; *P*=0.07 for BMS614-treated *u768* mutants versus DMSO-treated *u768* mutants). By 40 hpf, both BMS614-treated *gdf6a^u768^* mutant eyes and DMSO-treated sibling eyes contained similar GFP^+^ retinal volumes whereas DMSO-treated *gdf6a^u768^* mutant eyes contained substantially larger GFP^+^ retinal volumes (Fig. 7I,J,L; *P*=0.001 for BMS614-treated *u768* mutants versus DMSO-treated *u768* mutants).

Further suggesting that precocious neurogenesis underlies the *gdf6a* mutant phenotype, we found that mutant eyes contain a higher proportion of GFP^+^ neurons in the RGC layer by 80 hpf (Fig. 7M-O; Fig. S6) and more GFP^+^ cells in the inner nuclear layer, suggesting that more *atoh7*-positive cells are generated earlier (Fig. 7N, white arrows). Together, these observations suggest that RA can promote cell cycle exit and the production of *atoh7*-positive neurons in developing retinae.

## DISCUSSION

This study reveals that opposing activities of Gdf6a from the dorsal retina and RA from the ventral retina influence RPCs as they transition from proliferation to differentiation, ultimately regulating eye size. Our data demonstrate that in addition to previously demonstrated roles in DV patterning, the combined action of these pathways regulates when RPCs exit the cell cycle. Our genetic and pharmacological manipulations illustrate that Gdf6a-mediated inhibition of RA pathway activity modulates the timing of retinal neurogenesis and suggest that RA-mediated precocious differentiation of the RPC pool might underlie the microphthalmic phenotype in vertebrates carrying *GDF6* mutations.

### Gdf6a regulates retinal growth

Our study adds to others linking eye defects to abrogation of Gdf6 function in fish, mice and humans (Asai-Coakwell et al., 2007, 2013; den Hollander et al., 2010; Gosse and Baier, 2009; Hanel and Hensey, 2006), but the mechanisms connecting loss of *gdf6a* to microphthalmia have remained unclear. It has been suggested that apoptosis contributes to the small eye phenotype in *gdf6a* mutants (den Hollander et al., 2010; Gosse and Baier, 2009). Our study and another (French et al., 2013), provide evidence that microphthalmia occurs independently of cell death in *gdf6a* mutants. Pronounced differences in eye size are observed in *gdf6a^u768^* mutants, which exhibit levels of retinal apoptosis comparable to wild type, and in *gdf6a^u900^* embryos when apoptosis is blocked. Despite these differences in apoptosis, both *gdf6a* alleles exhibit similar changes in gene expression, proliferation and differentiation. Moreover, analyses of *gdf6a^u768^* mutants suggest that abnormal initiation of retinal DV patterning is not a prerequisite for *gdf6a*-linked microphthalmia.

### Gdf proteins regulate cell cycle exit and differentiation through various mechanisms

Members of the BMP family regulate cell fate, proliferation, differentiation, and apoptosis during embryonic development. As a result, BMPs and their antagonists have been implicated in control of tissue and organ size (Beites et al., 2009; Sartori et al., 2013). The presence of fewer proliferating cells and the accelerated expression of differentiation genes in the early retinae of *gdf6a* mutants suggest that the BMP-related protein Gdf6a is part of a genetic circuit that balances progenitor cell proliferation and differentiation to control eye size in zebrafish. In mammals, GDF11 and GDF8 (MSTN) balance proliferation and differentiation in different contexts. Both act as auto-inhibitory feedback signals to directly and reversibly regulate cell cycle exit of neuronal progenitors in the olfactory epithelium (Wu et al., 2003) and myoblasts (McPherron and Lee, 1997), respectively. GDF11 also modulates retinal size in mice but through a different mechanism. During early neurogenesis, expression of GDF11 and its inhibitor, follistatin, influence the timing of expression of *Atoh7* (also known as *Math5*) without affecting expression of cell cycle regulators (Kim et al., 2005). Despite diverse mechanisms by which these GDF proteins act, they promote similar consequences for timing of cell cycle exit and differentiation.

### Gdf6a-antagonised RA pathway activity regulates cell cycle exit and differentiation

Our study reveals that a key role for Gdf6a is to limit RA activity in RPCs. We show that enhancing RA activity accelerates the production of neurons from RPCs and this presumably depletes the pool of proliferative precursors. Such a role for RA is consistent with studies showing that RA promotes neuronal differentiation *in vitro* (reviewed by Janesick et al., 2015). In the vertebrate retina, RA can skew the fate of post-mitotic photoreceptor precursors towards rod and red-sensitive cone fates (Hyatt et al., 1996; Kelley et al., 1999; Mitchell et al., 2015; Stevens et al., 2011) and *in vitro* studies of oligodendrocyte precursors suggest that the timing of differentiation is modulated by retinoid signalling (Barres et al., 1994). Similarly, our data indicate that RA influences the timing of cell cycle exit and neuronal differentiation in the developing central retina and CMZ. Consequently, unlike other Gdfs that regulate timing of neurogenesis by promoting cell cycle exit (Gokoffski et al., 2011; Kim et al., 2005; Wu et al., 2003), our data suggest that Gdf6a is permissive for proliferation and, in balance with RA signalling, governs the timing of differentiation in the retina to preserve the pool of progenitors needed for multiple rounds of neurogenesis.

Although our data suggest that antagonism between Gdf6a and RA regulates RPC behaviours, Gdf6a could also interact with other signalling pathways to influence proliferation and differentiation. For instance, Fgf signalling maintains a subpopulation of RPCs in a proliferative state (Wong et al., 2015), and Hedgehog signalling is implicated in RPC proliferation and differentiation decisions (Borday et al., 2012; Locker et al., 2006; Masai et al., 2000). Cross-talk and integration between these pathways and BMP have been demonstrated in many diverse progenitor cell populations, including those in the developing neural tube (e.g. Horner and Caspary, 2011; Sasai et al., 2014).

### Intersection between morphogenetic and neurogenic effects of Gdf6a

In addition to the requirement for Gdf6a to establish and maintain dorsal retinal character, we suggest an additional role for Gdf6a during eye development. Our data suggest that Gdf6a modulates RA pathway activity, which in turn influences the probability of RPC cell cycle exit by regulating proliferative versus neurogenic divisions. Bolstering this idea, we observed enhanced and accelerated expression of the bHLH transcription factor-encoding gene *atoh7*, which is required for RGC production (Kay et al., 2001). Furthermore, we show that normal timing of *atoh7* expression is restored when RA pathway activity is downregulated in *gdf6a* mutants. Two observations from studies in medaka fish also support a connection between RA, *atoh7* expression, and eye size. First, a global analysis of the regulatory inputs driving retinal neurogenesis identified a potential positive role for RA upstream of *atoh7* expression (Souren et al., 2009), and, second, a study that expanded *atoh7* expression throughout the developing retina generated smaller eyes (Sinn et al., 2014).

How might an imbalance between RA and Gdf6a contribute to the small eyes in *gdf6a* mutants? We observe a smaller CMZ in mutant eyes as well as in eyes in which the RA pathway is activated early in development. Consequently, the failure of *gdf6a* eyes to grow robustly could be due to problems in both establishment of the CMZ and generation of neurons from the CMZ. Although studies have begun to examine the origins of the CMZ (Heermann et al., 2015; Kwan et al., 2012), how this stem cell niche is established and maintained is not clear. One possibility is that in *gdf6a* mutants, precocious differentiation of RPCs depletes the pool of progenitors that ultimately populate the CMZ, thus generating a smaller CMZ.

Coupled with the possibility of fewer stem and progenitor cells in the CMZ of *gdf6a* mutants, defective Gdf6a/retinoid signalling within the CMZ is likely to further limit eye growth by exhausting the stem cell/progenitor population. Many signalling pathways required during early eye development, including BMP and RA pathways, remain active within the CMZ (Harris and Perron, 1998; Sharma et al., 2005; Shawi and Serluca, 2008). Previous work from our laboratories supports a model for differentiation dynamics within the zebrafish CMZ with at least two sets of environmental signals coexisting. One set of signals promotes proliferation of stem and progenitor cells and the other limits proliferation of rapidly cycling precursors by encouraging differentiation (Cerveny et al., 2010). Although a molecular basis for this so-called ‘environmentally driven differentiation’ has yet to be established, our current work suggests that Gdf6a and RA are two secreted signals that establish an appropriate balance between proliferation and differentiation in the CMZ. Together, our results support a model in which dorsally secreted Gdf6a balances RA pathway activity, controlling the transition from proliferation to differentiation during eye growth.

## MATERIALS AND METHODS

### Zebrafish lines

Eggs were collected by natural spawning, raised at 28.5°C in either fish water or E3 embryo medium (Nüsslein-Volhard and Dahm, 2002) and staged according to Kimmel et al. (1995). Transgenic and mutant lines are listed in supplementary Materials and Methods. To prevent pigment formation, 0.003% phenylthiourea (PTU, Sigma) was added to the fish water between 20 and 24 hpf.

### ENU mutagenesis and eye screening

Mutagenesis was performed in wild-type male AB/TL fish by four rounds of 3 mM ENU treatment as previously described (Valdivia et al., 2011; van Eeden et al., 1999). Eyes of F3 larvae were screened for morphological abnormalities.

### Genetic mapping, cloning and genotyping

The *gdf6a^u768^* mutation was mapped by bulk segregant analysis with simple sequence length polymorphisms to LG16 (Talbot and Schier, 1999). Markers Z13555 and Z45043 flanked a ∼2 Mb interval containing *gdf6a*. *gdf6a^u768^* carriers were crossed to a reported *gdf6a* mutant line (*rdr^s327^*; Gosse and Baier, 2009) for complementation testing. The *gdf6a^u900^* mutation was in turn complementation tested with *gdf6a^u768^*. To identify the molecular nature of both mutations, we performed RT-PCR (SuperScript-II Reverse Transcriptase and random primers; Invitrogen) on RNA isolated from 3 dpf wild-type and *gdf6a^u768^* larvae (Trizol, Invitrogen), and PCR on genomic DNA obtained using the HotSHOT method (Meeker et al., 2007) for *gdf6a^u900^*. Amplicons were cloned into TOPO-TA vectors (Invitrogen) for sequencing. Oligonucleotides for cloning, sequencing and genotyping are listed in supplementary Materials and Methods.

### Microinjections

Capped histone H2B-red fluorescent protein fusion (H2B-RFP) mRNA was prepared using the mMessage mMachine RNA Synthesis Kit (Ambion) according to the manufacturer's instructions. One-cell-stage embryos resulting from *gdf6a^u900^* heterozygous in-crosses carrying the *Tg[atoh7:GFP]^cu2^* transgene were injected with 150 pg of mRNA.

### Histology

To prepare *in situ* hybridisation probes, DNA templates were generated by restriction digestion of plasmids carrying *atoh7*, *ccnd1*, *cdkn1c*, *nr2f5*, *aldh1a2*, *aldh1a3* and *cyp26a1* or by PCR from cDNA for *col15a1b*, *crabp2a* and *rdh10a*. For each template, the reverse strand oligonucleotide encodes a T3 polymerase priming site as well as the gene-specific sequence (Thisse and Thisse, 2008). All primers are provided in supplementary Materials and Methods. Digoxigenin-labelled RNA probes were transcribed using a DIG labelling kit (Roche) with the appropriate polymerases (Promega or NEB). Embryos were processed as previously described (Thisse and Thisse, 2008) and hybridisation signals were detected with anti-digoxigenin-AP antibody (1:3000; 11093274910, Roche) and NBT/BCIP substrate (1:3.5; Roche).

Apoptotic cell death was detected by terminal deoxynucleotidyl transferase-mediated dUTP nick end labelling (TUNEL). Embryos were fixed in 4% paraformaldehyde overnight and kept in methanol 100% until used. Following rehydration, embryos were treated following manufacturer instructions using the ApopTag kit (Millipore). An anti-digoxigenin-AP antibody (1:3000; Roche) was used for NBT/BCIP (Roche) detection.

For sectioning, embryos were fixed with 4% paraformaldehyde overnight at 4°C and then cryoprotected by sequential incubation in 15% and 30% sucrose dissolved in PBS supplemented with 0.5% Triton X-100 (PBST) for 12-16 h at 4°C. They were embedded in OCT, frozen on dry ice, and sectioned at 16-25 µm using a Leica cryostat. All immunostaining steps were performed at room temperature (∼22°C).

BrdU incorporation was performed at room temperature as previously described (Cerveny et al., 2010). Briefly, 1 nl pulses of 10 mg/ml BrdU in E3 were injected into the hearts of embryos anaesthetised with MS-222 and immobilised in 1% low melting point agarose dissolved in E3. After injection at defined time points, embryos were liberated from agarose and incubated in E3 until fixation with 4% paraformaldehyde.

Primary antibodies used were: chicken or mouse anti-GFP (ab13970 and ab1218, Abcam; 1:1000); rabbit anti-RFP (PM005, Medical & Biological Laboratories Co.; 1:2500); mouse anti-BrdU (3262F, Millipore; 1:200); rabbit anti-PH3 (06-570, Millipore; 1:400).

### Microscopy and image analysis

After *in situ* hybridisation or before immunostaining, embryo tails were genotyped and heads and/or dissected eyes were either imaged using a Nikon E1000 microscope equipped with DIC 20×0.5 NA and 40×1.15 NA objective lenses or subjected to immunohistochemistry. After immunohistochemistry or for time-lapse imaging of transgenic lines, sections or agarose-embedded embryos were imaged with Leica SPE (25×0.95 NA and 40×0.8 NA water immersion objectives) or Nikon A1+ (25×1.1 NA water immersion objective) confocal microscopes.

Digital images were processed with ImageJ and/or Imaris (Bitplane) software and compiled using Photoshop CS6 (Adobe). For some images, white balance was digitally adjusted using the exposure option in Photoshop CS6. For quantifying TUNEL^+^, PH3^+^ and PH3^+^/BrdU^+^ cells, images were blind-counted using ImageJ. To quantify the volume of retinae containing GFP^+^ cells, the fluorescence of each *z*-plane of confocal stacks (∼60 µm) was thresholded, images compiled and a contour surface drawn for both the entire eye and the fluorescent portion using the surface contour feature and either manual selection (for the entire eye) or auto selection (for GFP fluorescence) of Imaris. For eye and section size measurements, images taken from a lateral view at fixed magnification were opened in ImageJ. The freehand selection tool was used to select to outline the circumference of each eye and then the measure function was used to calculate the area. Data were exported to R or Prism (GraphPad) for statistical analysis and graphing. Either permutation testing with 10,000 shuffles or Student's *t*-tests with 5% cut-offs were used as indicated in the Results and figure legends. See supplementary Materials and Methods for details of permutation testing.

### Time-lapse imaging

Confocal time-lapse imaging was performed on a Leica SP8 confocal microscope in an air chamber heated to 28.5°C. Once anaesthetised with MS-222 (Sigma), embryos were immobilised in 1.2% low melting point agarose (Sigma) and imaged from a lateral view using water immersion objectives. *Z*-stacks were collected at intervals of 20 min for 9 h, beginning at 28.5 hpf.

### Pharmacological manipulation of apoptotic or RA pathways

To block apoptosis, embryos were incubated with either 200 or 400 µM of the pan-caspase inhibitor Z-VAD-FMK (Promega) from 14 to 31 hpf (Williams et al., 2000). The drug was washed out and embryos were either fixed or incubated in fish water until 3 dpf and then fixed.

To activate RA pathway activity, embryos were soaked in 25 nM AM580 (Sigma), a pan-retinoic acid receptor (RAR) agonist, beginning at 24 hpf for the times indicated in figure legends. To inhibit the pathway, embryos were soaked in either 15 µM BMS493 (Sigma), an RARα inverse agonist, or 15 µM BMS614 (Tocris), an RARα antagonist, for 14-36 h from the 12-14 somite stage, which is after initial dorsal-ventral patterning of the eye and brain. After drug treatments, embryos were washed twice with embryo medium and then incubated until fixation with 4% paraformaldehyde at 28, 40 or 60 hpf. For optical manipulation of RA activity in the retina, *Tg[RARE:YFP]^id1^* embryos were soaked in 5 nM 13-cisRA (Sigma; Xu et al., 2012) at 24 hpf for 1 h in the dark and then mounted in 1.2% low melting point agarose. Photoactivation was performed with a single pulse of UV light (360-375 nm) illuminating the eye for 30 s, using a Zeiss 510 NLO two-photon microscope with 5 mW of power. Embryos were fixed at 33 hpf in 4% paraformaldehyde and immunostained for GFP.

### Quantitative real-time PCR

Quantitative RT-PCR was performed with total RNA extracted from dissected fixed retinae at 28 hpf and 3 dpf. Briefly, embryos were fixed in 4% paraformaldehyde overnight and transferred to RNAse-free PBS, after two washes for 5 min each. Embryo tails were genotyped using KASP assays (Kettleborough et al., 2013). Heads were pooled by genotype and retinae dissected manually using insect pins. RNA was extracted using RecoverAll Total Nucleic Acid Isolation Kit for FFPE (Ambion) from 40 retinas for each condition (wild-type sibling versus mutant at each time point). RNA quality control was performed with the Experion LabChip (Bio-Rad). cDNA was synthesised and amplified with the Transplex Whole Transcriptome Amplification Kit (Sigma), and quantified using a Nanodrop 2000c. Quantitect primers (Qiagen) were used to amplify *col15a1b* (QT02215941), *ccnd1* (QT02178519), *atoh7* (QT02188459), *nr2f5* (QT02125424), *aldh1a3* (QT02111613), *crx1* (QT02229584), *cdkn1c* (QT02052253) and *β-actin* (QT02174907). Real-time PCR was performed on a BioRad iCycler using GoTaq qPCR Master Mix (Promega). Fold change in RNA levels was calculated using the ΔΔCt method, and expression normalised to *β-actin* levels (Livak and Schmittgen, 2001).

## References

[DEV130922C1] Agathocleous M. and Harris W. A. (2009). From progenitors to differentiated cells in the vertebrate retina. *Annu. Rev. Cell Dev. Biol.* 25, 45-69. 10.1146/annurev.cellbio.042308.113259 19575661

[DEV130922C2] Asai-Coakwell M., French C. R., Berry K. M., Ye M., Koss R., Somerville M., Mueller R., van Heyningen V., Waskiewicz A. J. and Lehmann O. J. (2007). GDF6, a novel locus for a spectrum of ocular developmental anomalies. *Am. J. Hum. Genet.* 80, 306-315. 10.1086/511280 17236135 PMC1785352

[DEV130922C3] Asai-Coakwell M., March L., Dai X. H., DuVal M., Lopez I., French C. R., Famulski J., De Baere E., Francis P. J., Sundaresan P. et al. (2013). Contribution of growth differentiation factor 6-dependent cell survival to early-onset retinal dystrophies. *Hum. Mol. Genet.* 22, 1432-1442. 10.1093/hmg/dds560 23307924

[DEV130922C4] Barres B. A., Lazar M. A. and Raff M. C. (1994). A novel role for thyroid hormone, glucocorticoids and retinoic acid in timing oligodendrocyte development. *Development* 120, 1097-1108. 8026323 10.1242/dev.120.5.1097

[DEV130922C5] Bassett E. A. and Wallace V. A. (2012). Cell fate determination in the vertebrate retina. *Trends Neurosci.* 35, 565-573. 10.1016/j.tins.2012.05.004 22704732

[DEV130922C6] Beites C. L., Hollenbeck P. L. W., Kim J., Lovell-Badge R., Lander A. D. and Calof A. L. (2009). Follistatin modulates a BMP autoregulatory loop to control the size and patterning of sensory domains in the developing tongue. *Development* 136, 2187-2197. 10.1242/dev.030544 19474151 PMC2729339

[DEV130922C7] Borday C., Cabochette P., Parain K., Mazurier N., Janssens S., Tran H. T., Sekkali B., Bronchain O., Vleminckx K., Locker M. et al. (2012). Antagonistic cross-regulation between Wnt and Hedgehog signalling pathways controls post-embryonic retinal proliferation. *Development* 139, 3499-3509. 10.1242/dev.079582 22899850

[DEV130922C8] Cai A. Q., Radtke K., Linville A., Lander A. D., Nie Q. and Schilling T. F. (2012). Cellular retinoic acid-binding proteins are essential for hindbrain patterning and signal robustness in zebrafish. *Development* 139, 2150-2155. 10.1242/dev.077065 22619388 PMC3357909

[DEV130922C9] Centanin L., Hoeckendorf B. and Wittbrodt J. (2011). Fate restriction and multipotency in retinal stem cells. *Cell Stem Cell* 9, 553-562. 10.1016/j.stem.2011.11.004 22136930

[DEV130922C10] Cepko C. (2014). Intrinsically different retinal progenitor cells produce specific types of progeny. *Nat. Rev. Neurosci.* 15, 615-627. 10.1038/nrn3767 25096185

[DEV130922C11] Cepko C. L., Austin C. P., Yang X., Alexiades M. and Ezzeddine D. (1996). Cell fate determination in the vertebrate retina. *Proc. Natl. Acad. Sci. USA* 93, 589-595. 10.1073/pnas.93.2.589 8570600 PMC40096

[DEV130922C12] Cerveny K. L., Cavodeassi F., Turner K. J., de Jong-Curtain T. A., Heath J. K. and Wilson S. W. (2010). The zebrafish flotte lotte mutant reveals that the local retinal environment promotes the differentiation of proliferating precursors emerging from their stem cell niche. *Development* 137, 2107-2115. 10.1242/dev.047753 20504962 PMC2882130

[DEV130922C13] Cerveny K. L., Varga M. and Wilson S. W. (2012). Continued growth and circuit building in the anamniote visual system. *Dev. Neurobiol.* 72, 328-345. 10.1002/dneu.20917 21563317

[DEV130922C14] den Hollander A. I., Biyanwila J., Kovach P., Bardakjian T., Traboulsi E. I., Ragge N. K., Schneider A. and Malicki J. (2010). Genetic defects of GDF6 in the zebrafish out of sight mutant and in human eye developmental anomalies. *BMC Genet.* 11, 102 10.1186/1471-2156-11-102 21070663 PMC2992036

[DEV130922C15] French C. R., Erickson T., French D. V., Pilgrim D. B. and Waskiewicz A. J. (2009). Gdf6a is required for the initiation of dorsal–ventral retinal patterning and lens development. *Dev. Biol.* 333, 37-47. 10.1016/j.ydbio.2009.06.018 19545559

[DEV130922C16] French C. R., Stach T. R., March L. D., Lehmann O. J. and Waskiewicz A. J. (2013). Apoptotic and proliferative defects characterize ocular development in a microphthalmic BMP model. *Invest. Ophthalmol. Vis. Sci.* 54, 4636-4647. 10.1167/iovs.13-11674 23737474

[DEV130922C17] Germain P., Gaudon C., Pogenberg V., Sanglier S., Van Dorsselaer A., Royer C. A., Lazar M. A., Bourguet W. and Gronemeyer H. (2009). Differential action on coregulator interaction defines inverse retinoid agonists and neutral antagonists. *Chem. Biol.* 16, 479-489. 10.1016/j.chembiol.2009.03.008 19477412

[DEV130922C18] Gianni M., Li Calzi M., Terao M., Guiso G., Caccia S., Barbui T., Rambaldi A. and Garattini E. (1996). AM580, a stable benzoic derivative of retinoic acid, has powerful and selective cyto-differentiating effects on acute promyelocytic leukemia cells. *Blood* 87, 1520-1531. 8608243

[DEV130922C19] Gokoffski K. K., Wu H.-H., Beites C. L., Kim J., Kim E. J., Matzuk M. M., Johnson J. E., Lander A. D. and Calof A. L. (2011). Activin and GDF11 collaborate in feedback control of neuroepithelial stem cell proliferation and fate. *Development* 138, 4131-4142. 10.1242/dev.065870 21852401 PMC3171217

[DEV130922C20] Gonzalez-Nunez V., Nocco V. and Budd A. (2010). Characterization of drCol 15a1b: a novel component of the stem cell niche in the zebrafish retina. *Stem Cells* 28, 1399-1411. 10.1002/stem.461 20549708

[DEV130922C21] Gosse N. J. and Baier H. (2009). An essential role for Radar (Gdf6a) in inducing dorsal fate in the zebrafish retina. *Proc. Natl. Acad. Sci. USA* 106, 2236-2241. 10.1073/pnas.0803202106 19164594 PMC2650138

[DEV130922C22] Hanel M. L. and Hensey C. (2006). Eye and neural defects associated with loss of GDF6. *BMC Dev. Biol.* 6, 43 10.1186/1471-213X-6-43 17010201 PMC1609107

[DEV130922C23] Harris W. A. (1997). Cellular diversification in the vertebrate retina. *Curr. Opin. Genet. Dev.* 7, 651-658. 10.1016/S0959-437X(97)80013-5 9388782

[DEV130922C24] Harris W. A. and Perron M. (1998). Molecular recapitulation: the growth of the vertebrate retina. *Int. J. Dev. Biol.* 42, 299-304. 9654012

[DEV130922C25] Heermann S., Schütz L., Lemke S., Krieglstein K. and Wittbrodt J. (2015). Eye morphogenesis driven by epithelial flow into the optic cup facilitated by modulation of bone morphogenetic protein. *eLife* 4, 373 10.7554/eLife.05216 PMC4337729 25719386

[DEV130922C26] Hernandez-Bejarano M., Gestri G., Spawls L., Nieto-Lopez F., Picker A., Tada M., Brand M., Bovolenta P., Wilson S. W. and Cavodeassi F. (2015). Opposing Shh and Fgf signals initiate nasotemporal patterning of the zebrafish retina. *Development* 142, 3933-3942. 10.1242/dev.125120 26428010 PMC4712879

[DEV130922C27] Horner V. L. and Caspary T. (2011). Disrupted dorsal neural tube BMP signaling in the cilia mutant Arl13bhnn stems from abnormal Shh signaling. *Dev. Biol.* 355, 43-54. 10.1016/j.ydbio.2011.04.019 21539826 PMC3119544

[DEV130922C28] Hyatt G. A., Schmitt E. A., Fadool J. M. and Dowling J. E. (1996). Retinoic acid alters photoreceptor development in vivo. *Proc. Natl. Acad. Sci. USA* 93, 13298-13303. 10.1073/pnas.93.23.13298 8917585 PMC24087

[DEV130922C29] Isken A., Golczak M., Oberhauser V., Hunzelmann S., Driever W., Imanishi Y., Palczewski K. and von Lintig J. (2008). RBP4 disrupts vitamin A uptake homeostasis in a STRA6-deficient animal model for Matthew-Wood syndrome. *Cell Metab.* 7, 258-268. 10.1016/j.cmet.2008.01.009 18316031 PMC2561276

[DEV130922C30] Janesick A., Wu S. C. and Blumberg B. (2015). Retinoic acid signaling and neuronal differentiation. *Cell. Mol. Life Sci.* 72, 1559-1576. 10.1007/s00018-014-1815-9 25558812 PMC11113123

[DEV130922C31] Kay J. N., Finger-Baier K. C., Roeser T., Staub W. and Baier H. (2001). Retinal ganglion cell genesis requires lakritz, a zebrafish atonal homolog. *Neuron* 30, 725-736. 10.1016/S0896-6273(01)00312-9 11430806

[DEV130922C32] Kelley M. W., Williams R. C., Turner J. K., Creech-Kraft J. M. and Reh T. A. (1999). Retinoic acid promotes rod photoreceptor differentiation in rat retina in vivo. *Neuroreport* 10, 2389-2394. 10.1097/00001756-199908020-00031 10439469

[DEV130922C33] Kettleborough R. N. W., Busch-Nentwich E. M., Harvey S. A., Dooley C. M., de Bruijn E., van Eeden F., Sealy I., White R. J., Herd C., Nijman I. J. et al. (2013). A systematic genome-wide analysis of zebrafish protein-coding gene function. *Nature* 496, 494-497. 10.1038/nature11992 23594742 PMC3743023

[DEV130922C34] Kim J., Wu H.-H., Lander A. D., Lyons K. M., Matzuk M. M. and Calof A. L. (2005). GDF11 controls the timing of progenitor cell competence in developing retina. *Science* 308, 1927-1930. 10.1126/science.1110175 15976303

[DEV130922C35] Kimmel C. B., Ballard W. W., Kimmel S. R., Ullmann B. and Schilling T. F. (1995). Stages of embryonic development of the zebrafish. *Dev. Dyn.* 203, 253-310. 10.1002/aja.1002030302 8589427

[DEV130922C36] Kruse-Bend R., Rosenthal J., Quist T. S., Veien E. S., Fuhrmann S., Dorsky R. I. and Chien C. B. (2012). Extraocular ectoderm triggers dorsal retinal fate during optic vesicle evagination in zebrafish. *Dev. Biol.* 371, 57-65. 10.1016/j.ydbio.2012.08.004 22921921 PMC3455121

[DEV130922C37] Kwan K. M., Otsuna H., Kidokoro H., Carney K. R., Saijoh Y. and Chien C.-B. (2012). A complex choreography of cell movements shapes the vertebrate eye. *Development* 139, 359-372. 10.1242/dev.071407 22186726 PMC3243097

[DEV130922C38] Livak K. J. and Schmittgen T. D. (2001). Analysis of relative gene expression data using real-time quantitative PCR and the 2(-Delta Delta C(T)) Method. *Methods* 25, 402-408. 10.1006/meth.2001.1262 11846609

[DEV130922C39] Locker M., Agathocleous M., Amato M. A., Parain K., Harris W. A. and Perron M. (2006). Hedgehog signaling and the retina: insights into the mechanisms controlling the proliferative properties of neural precursors. *Genes Dev.* 20, 3036-3048. 10.1101/gad.391106 17079690 PMC1620016

[DEV130922C40] Love C. E. and Prince V. E. (2012). Expression and retinoic acid regulation of the zebrafish nr2f orphan nuclear receptor genes. *Dev. Dyn.* 241, 1603-1615. 10.1002/dvdy.23838 22836912 PMC3459307

[DEV130922C41] Lupo G., Gestri G., O'Brien M., Denton R. M., Chandraratna R. A. S., Ley S. V., Harris W. A. and Wilson S. W. (2011). Retinoic acid receptor signaling regulates choroid fissure closure through independent mechanisms in the ventral optic cup and periocular mesenchyme. *Proc. Natl. Acad. Sci. USA* 108, 8698-8703. 10.1073/pnas.1103802108 21555593 PMC3102374

[DEV130922C42] Marsh-Armstrong N., McCaffery P., Gilbert W., Dowling J. E. and Drager U. C. (1994). Retinoic acid is necessary for development of the ventral retina in zebrafish. *Proc. Natl. Acad. Sci. USA* 91, 7286-7290. 10.1073/pnas.91.15.7286 8041782 PMC44384

[DEV130922C43] Masai I., Stemple D. L., Okamoto H. and Wilson S. W. (2000). Midline signals regulate retinal neurogenesis in zebrafish. *Neuron* 27, 251-263. 10.1016/S0896-6273(00)00034-9 10985346

[DEV130922C44] McPherron A. C. and Lee S.-J. (1997). Double muscling in cattle due to mutations in the myostatin gene. *Proc. Natl. Acad. Sci. USA* 94, 12457-12461. 10.1073/pnas.94.23.12457 9356471 PMC24998

[DEV130922C45] Meeker N. D., Hutchinson S. A., Ho L. and Trede N. S. (2007). Method for isolation of PCR-ready genomic DNA from zebrafish tissues. *Biotechniques* 43, 610-614. 10.2144/000112619 18072590

[DEV130922C46] Mitchell D. M., Stevens C. B., Frey R. A., Hunter S. S., Ashino R., Kawamura S. and Stenkamp D. L. (2015). Retinoic acid signaling regulates differential expression of the tandemly-duplicated long wavelength-sensitive cone opsin genes in zebrafish. *PLoS Genet.* 11, e1005483 10.1371/journal.pgen.1005483 26296154 PMC4546582

[DEV130922C47] Neumann C. J. and Nuesslein-Volhard C. (2000). Patterning of the zebrafish retina by a wave of sonic hedgehog activity. *Science* 289, 2137-2139. 10.1126/science.289.5487.2137 11000118

[DEV130922C48] Nüsslein-Volhard C. and Dahm R. (2002). Zebrafish: a practical approach. Oxford, UK: Oxford University Press.

[DEV130922C49] Ohnuma S.-i., Philpott A., Wang K., Holt C. E. and Harris W. A. (1999). p27Xic1, a Cdk inhibitor, promotes the determination of glial cells in Xenopus retina. *Cell* 99, 499-510. 10.1016/S0092-8674(00)81538-X 10589678

[DEV130922C50] Perz-Edwards A., Hardison N. L. and Linney E. (2001). Retinoic acid-mediated gene expression in transgenic reporter zebrafish. *Dev. Biol.* 229, 89-101. 10.1006/dbio.2000.9979 11133156

[DEV130922C51] Rappaport D. H. (2006). Retinal development. In *Retinal Development* (ed. Sernagor E., Eglen S., Harris W. A. and Wong R.), pp. 30-58. Cambridge University Press.

[DEV130922C52] Raymond P. A., Barthel L. K., Bernardos R. L. and Perkowski J. J. (2006). Molecular characterization of retinal stem cells and their niches in adult zebrafish. *BMC Dev. Biol.* 6, 36 10.1186/1471-213X-6-36 16872490 PMC1564002

[DEV130922C53] Rissi M., Wittbrodt J., Délot E., Naegeli M. and Rosa F. M. (1995). Zebrafish Radar: a new member of the TGF-beta superfamily defines dorsal regions of the neural plate and the embryonic retina. *Mech. Dev.* 49, 223-234. 10.1016/0925-4773(94)00320-M 7734395

[DEV130922C54] Sartori R., Schirwis E., Blaauw B., Bortolanza S., Zhao J., Enzo E., Stantzou A., Mouisel E., Toniolo L., Ferry A. et al. (2013). BMP signaling controls muscle mass. *Nat. Genet.* 45, 1309-1318. 10.1038/ng.2772 24076600

[DEV130922C55] Sasai N., Kutejova E. and Briscoe J. (2014). Integration of signals along orthogonal axes of the vertebrate neural tube controls progenitor competence and increases cell diversity. *PLoS Biol.* 12, e1001907 10.1371/journal.pbio.1001907 25026549 PMC4098999

[DEV130922C56] Schmidt R., Strähle U. and Scholpp S. (2013). Neurogenesis in zebrafish – from embryo to adult. *Neural Dev.* 8, 3 10.1186/1749-8104-8-3 23433260 PMC3598338

[DEV130922C57] Sharma M. K., Saxena V., Liu R.-Z., Thisse C., Thisse B., Denovan-Wright E. M. and Wright J. M. (2005). Differential expression of the duplicated cellular retinoic acid-binding protein 2 genes (crabp2a and crabp2b) during zebrafish embryonic development. *Gene Expr. Patterns* 5, 371-379. 10.1016/j.modgep.2004.09.010 15661643

[DEV130922C58] Shawi M. and Serluca F. C. (2008). Identification of a BMP7 homolog in zebrafish expressed in developing organ systems. *Gene Expr. Patterns* 8, 369-375. 10.1016/j.gep.2008.05.004 18602348

[DEV130922C59] Shen Y.-c. and Raymond P. A. (2004). Zebrafish cone-rod (crx) homeobox gene promotes retinogenesis. *Dev. Biol.* 269, 237-251. 10.1016/j.ydbio.2004.01.037 15081370

[DEV130922C60] Sinn R., Peravali R., Heermann S. and Wittbrodt J. (2014). Differential responsiveness of distinct retinal domains to Atoh7. *Mech. Dev.* 133, 218-229. 10.1016/j.mod.2014.08.002 25151399 PMC4232737

[DEV130922C61] Souren M., Martinez-Morales J., Makri P., Wittbrodt B. and Wittbrodt J. (2009). A global survey identifies novel upstream components of the Ath5 neurogenic network. *Genome Biol.* 10, R92 10.1186/gb-2009-10-9-r92 19735568 PMC2768981

[DEV130922C62] Stenkamp D. L. (2007). Neurogenesis in the fish retina. *Int. Rev. Cytol.* 259, 173-224. 10.1016/S0074-7696(06)59005-9 17425942 PMC2897061

[DEV130922C63] Stevens C. B., Cameron D. A. and Stenkamp D. L. (2011). Plasticity of photoreceptor-generating retinal progenitors revealed by prolonged retinoic acid exposure. *BMC Dev. Biol.* 11, 51 10.1186/1471-213X-11-51 21878117 PMC3189157

[DEV130922C64] Talbot W. S. and Schier A. F. (1999). Positional cloning of mutated zebrafish genes. *Methods Cell Biol.* 60, 259-286. 10.1016/S0091-679X(08)61905-6 9891342

[DEV130922C65] Thisse C. and Thisse B. (2008). High-resolution in situ hybridization to whole-mount zebrafish embryos. *Nat. Protoc.* 3, 59-69. 10.1038/nprot.2007.514 18193022

[DEV130922C66] Urbán N. and Guillemot F. (2014). Neurogenesis in the embryonic and adult brain: same regulators, different roles. *Front. Cell. Neurosci.* 8, 396 10.3389/fncel.2014.00396 25505873 PMC4245909

[DEV130922C67] Valdivia L. E., Young R. M., Hawkins T. A., Stickney H. L., Cavodeassi F., Schwarz Q., Pullin L. M., Villegas R., Moro E., Argenton F. et al. (2011). Lef1-dependent Wnt/beta-catenin signalling drives the proliferative engine that maintains tissue homeostasis during lateral line development. *Development* 138, 3931-3941. 10.1242/dev.062695 21862557 PMC3160090

[DEV130922C68] van Eeden F. J. M., Granato M., Odenthal J. and Haffter P. (1999). Developmental mutant screens in the zebrafish. *Methods Cell Biol.* 60, 21-41. 10.1016/S0091-679X(08)61892-0 9891329

[DEV130922C69] Vitorino M., Jusuf P. R., Maurus D., Kimura Y., Higashijima S.-i. and Harris W. A. (2009). Vsx2 in the zebrafish retina: restricted lineages through derepression. *Neural Dev.* 4, 14 10.1186/1749-8104-4-14 19344499 PMC2683830

[DEV130922C70] Williams J. A., Barrios A., Gatchalian C., Rubin L., Wilson S. W. and Holder N. (2000). Programmed cell death in zebrafish rohon beard neurons is influenced by TrkC1/NT-3 signaling. *Dev. Biol.* 226, 220-230. 10.1006/dbio.2000.9860 11023682

[DEV130922C71] Williams L. A., Bhargav D. and Diwan A. D. (2008). Unveiling the bmp13 enigma: redundant morphogen or crucial regulator? *Int. J. Biol. Sci.* 4, 318-329. 10.7150/ijbs.4.318 18797508 PMC2536705

[DEV130922C72] Wong L., Power N., Miles A. and Tropepe V. (2015). Mutual antagonism of the paired-type homeobox genes, vsx2 and dmbx1, regulates retinal progenitor cell cycle exit upstream of ccnd1 expression. *Dev. Biol.* 402, 216-228. 10.1016/j.ydbio.2015.03.020 25872183

[DEV130922C73] Wu H.-H., Ivkovic S., Murray R. C., Jaramillo S., Lyons K. M., Johnson J. E. and Calof A. L. (2003). Autoregulation of neurogenesis by GDF11. *Neuron* 37, 197-207. 10.1016/S0896-6273(02)01172-8 12546816

[DEV130922C74] Xu L., Feng Z., Sinha D., Ducos B., Ebenstein Y., Tadmor A. D., Gauron C., Le Saux T., Lin S., Weiss S. et al. (2012). Spatiotemporal manipulation of retinoic acid activity in zebrafish hindbrain development via photo-isomerization. *Development* 139, 3355-3362. 10.1242/dev.077776 22874920

[DEV130922C75] Yang X.-J. (2004). Roles of cell-extrinsic growth factors in vertebrate eye pattern formation and retinogenesis. *Semin. Cell Dev. Biol.* 15, 91-103. 10.1016/j.semcdb.2003.09.004 15036212 PMC7048382

